# Wire Arc Additive Manufactured Mild Steel and Austenitic Stainless Steel Components: Microstructure, Mechanical Properties and Residual Stresses

**DOI:** 10.3390/ma15207094

**Published:** 2022-10-12

**Authors:** Kasireddy Usha Rani, Rajiv Kumar, Manas M. Mahapatra, Rahul S. Mulik, Aleksandra Świerczyńska, Dariusz Fydrych, Chandan Pandey

**Affiliations:** 1 School of Mechanical Sciences, Indian Institute of Technology, Bhubaneswar 752050, India; 2 Mechanical and Industrial Engineering Department, Indian Institute of Technology Roorkee, Roorkee 247667, India; 3 Institute of Manufacturing and Materials Technology, Faculty of Mechanical Engineering and Ship Technology, Gdańsk University of Technology, Gabriela Narutowicza Street 11/12, 80-233 Gdańsk, Poland; 4 Department of Mechanical Engineering, Indian Institute of Technology Jodhpur N.H. 62, Nagaur Road Karwar, Jodhpur 342037, India

**Keywords:** mild steel, austenitic stainless steel, wire arc additive manufacturing, gas metal arc welding, residual stresses, strain relaxation method

## Abstract

Wire arc additive manufacturing (WAAM) is an additive manufacturing process based on the arc welding process in which wire is melted by an electric arc and deposited layer by layer. Due to the cost and rate benefits over powder-based additive manufacturing technologies and other alternative heat sources such as laser and electron beams, the process is currently receiving much attention in the industrial production sector. The gas metal arc welded (GMAW) based WAAM process provides a higher deposition rate than other methods, making it suitable for additive manufacturing. The fabrication of mild steel (G3Si1), austenitic stainless steel (SS304), and a bimetallic sample of both materials were completed successfully using the GMAW based WAAM process. The microstructure characterization of the developed sample was conducted using optical and scanning electron microscopes. The interface reveals two discrete zones of mild steel and SS304 deposits without any weld defects. The hardness profile indicates a drastic increase in hardness near the interface, which is attributed to chromium migration from the SS304. The toughness of the sample was tested based on the Charpy Impact (ASTM D6110) test. The test reveals isotropy in both directions. The tensile strength of samples deposited by the WAAM technique measured slightly higher than the standard values of weld filament. The deep hole drilling (DHD) method was used to measure the residual stresses, and it was determined that the stresses are compressive in the mild steel portion and tensile in austenitic stainless steel portion, and that they vary throughout the thickness due to variation in the cooling rate at the inner and outer surfaces.

## 1. Introduction

Over the past two decades, additive manufacturing (AM) has seen rapid development in industry to produce the complicated shape machine parts and die tools. The method involves either powder bed fusion (PBF) or direct energy deposition (DED). The paste extruding deposition (PED) process is also called selective laser melting and involves the melting and sintering of metallic powder and at the end of their accumulation. The method is used mainly for selective laser melting. The surface produced using PED has good dimensional accuracy and surface roughness compared with DED, but DED is still preferred over the PBF process as the metal deposition rate is low in PBF [1]. The most commonly used DED method is wire and arc-based additive manufacturing (WAAM) which utilizes arc welding technology. The process involves the melting of the filler wire using electric discharge and then deposition over the substrate material [2]. The WAAM process directly and locally add the material to molten pool. Li et al. [3,4] reviewed the various types of heat sources available in the WAAM process and reported the advantages and limitations of every source. Metal inert gas welding (MIG), tungsten inert gas welding (TIG), and plasma gas welding (PAW) are the major heat sources available for the WAAM process, and among these, the MIG process is easier and more convenient because of its direct-feeding spool of welding wire coaxial with the welding torch and process controlling [1]. In the MIG process, metal transfer occurs in five modes: globular, short-circuiting, spray, pulsed-spray, and cold metal transfer (CMT). Among these five modes of transfer, CMT provides a higher deposition rate, arc stability, and fine bead geometry [5]. Further research shows that CMT with the arc mode of pulse advance (CMT-PADV) is most suitable for the WAAM process as it can produce a wall with minimum porosity [6]. The TIG process uses a non-consumable tungsten electrode, and the wire is melted by the arc generated between the electrode and base material. In the PAW process, heat generated by the plasma arc is utilized to melt the wire. Paskual et al. [7] demonstrated that parts produced by the TIG process exhibited better mechanical properties and a higher percentage of elongation over the MIG and PAW processes. This improvement can be explained as the result of reduced heat input in the TIG process. In addition, homogeneity in the properties can also be observed irrespective of direction.

Among the three processes, gas metal arc welding (GMAW) provides a higher deposition rate, ultimately decreasing production time and cost. However, high heat input in GMAW has been found to have an adverse effect on weld bead geometry. The high heat input also results in high residual stresses and distortion. It is important to control the heat accumulation to reduce the residual stress generation. Heat accumulation can be controlled by optimizing the process parameters [8]. Intermittent cooling was introduced by Li et al. [9] to reduce the heat accumulation and they observed a 56.8% reduction in bead width inaccuracy. Heat input during the process also affects the microstructure evolution and, ultimately, the mechanical properties. Chen et al. [10,11] observed that the microstructure of gas metal arc additive manufacturing (GMA-AM) deposited 316L consists of δ, γ, and σ phases with different morphologies at different positions. Van et al. [12] deposited 308L stainless steel using the GMAW process and studied the microstructure evolution and mechanical properties. Anisotropy in tensile properties is observed in thin-walled components deposited by GMAW, and the same results were also observed by Paskual et al. [7] for AISI 316L and Ti6Al4V. Controlled short-circuiting metal transfer mode provides 16% reduction in heat input compared with the conventional GMAW process [13]. An increase in tensile strength and percentage elongation are also observed as a result of increased travel speed. 

Different materials can be deposited locally at the molten pool via the WAAM method, resulting in a mixture or gradient of physical and chemical properties. Dissimilar material deposition can be linked to dissimilar material welding. The joint interface strength is a crucial consideration for properly depositing dissimilar material, just as it is for dissimilar material welding. Mishra et al. [14] conducted comparative research on dissimilar metal joining of stainless steel and mild steel (MS) using TIG and MIG welding. It was observed that TIG welding is more efficient than MIG welding due to the low porosity in the dissimilar joining and the low amount of carbon precipitation. Rashid et al. [15] discussed the metallurgical properties of 316L stainless steel cladding on a mild steel substrate. Along the track laid, a decrease in the dilution content was found towards the end of the track. For different clad thicknesses, variation in hardness was also measured in the deposited tracks. Minute cracks and porosities were also observed due to residual thermal stresses. Using the WAAM process, Abe and Sasahara [16] experimented with bimetallic deposition of stainless steel and nickel-based alloys. The bond strength was comparable with the YS308L and Ni6082 weld materials. They tested adding a deposit of Ni6082 the surface of the structure and adding YS308L to the inner structure, which resulted in a considerable increase in heat and corrosion resistance. Wu et al. [17] employed an interweaving deposition strategy to boost the bond strength in steel-nickel bimetallic components and found that average tensile strength was higher than that of the feedstock material, which was attributed to the interlocking microstructure. In another study, bimetallic components of low carbon steel and 316L austenitic stainless steel were deposited by the WAAM process [18]. Microstructural observation showed a defect free area near the interface, and the tensile test results showed the failure from low carbon steel side because of the low strength. The hardness plot indicated an increase in hardness near the interface, which was attributed to the diffusion of Cr from stainless steel to the low carbon steel side. It has been observed that heat treatment has a significant effect on tensile strength and percentage elongation of WAAM-deposited bimetallic components. Ahsan et al. [19] observed an increase in ultimate tensile strength and elongation of 35% and 250%, respectively, after heat treatment of a bimetallic additively-manufactured structure. Residual stresses and distortion caused by repeated melting and solidification are key constraints of the WAAM process since they can reduce the component strength and induce dimensional variation. An experimental study conducted by Wu et al. [8] shows that substrate preheat temperature and heat input amount highly affects the residual stress generation. Increasing the preheat temperature and decreasing the heat input makes it possible to minimize residual stresses. It is critical to investigate the residual stress generation and variance in WAAM manufactured components. 

This work focused on the deposition of bimetallic structures using the GMAW WAAM process. The distribution of residual stress was experimentally determined based on the deep hole drilling (DHD) method. The hardness and strength of the fabricated structure were determined by conducting the Brinell and Charpy impact tests. Tensile strength was measured using the relation between hardness and tensile strength in both directions. 

## 2. Materials and Methods

### 2.1. Experimental Setup

**Figure 1:**
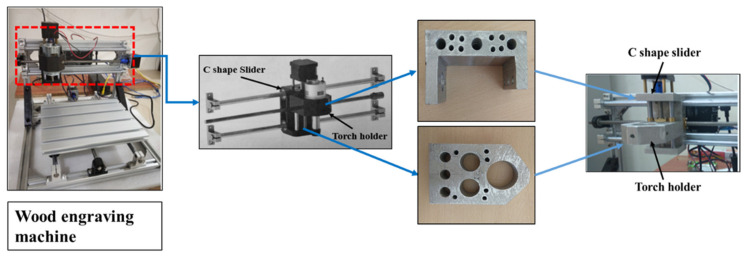
Replacement of wood engraving machine parts with metal parts.

A low-cost wood engraving machine was converted into a metal 3D printer to lower the cost (Figure 1). This machine is built with plastic parts; as they cannot withstand the high temperature during metal deposition, they were replaced by metal parts. These metal parts were made of 6061 aluminum alloy using sand casting to reduce the overall load on the stepper motor that came with the machine.

**Figure 2:**
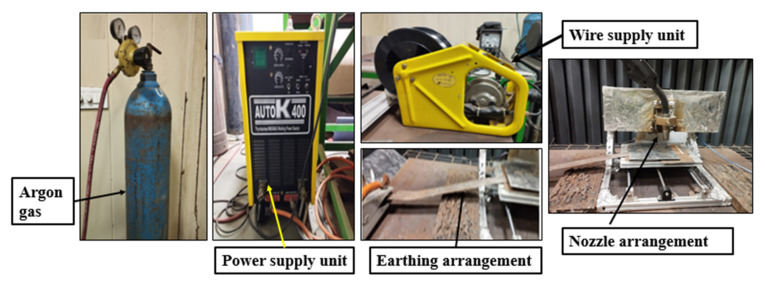
Experimental setup.

The casted part (Figure 1) is made of lightweight 6061 aluminium alloy [20]. Table 1 shows the composition of the alloy. Machining the casted parts was performed to obtain the desired shape and dimensions of the final parts. A misrun defect was observed in the casted parts because of the larger sizes of the parts. The cavity formed due to the misrun was filled later by pouring molten metal. The entire machine was wrapped with aluminium foil to protect it from welding spatter. Figure 2 shows the components of the experimental setup.

### 2.2. Materials and Experiment Conditions

A mild steel plate (S235JR) with dimensions of 100 mm × 100 mm × 3 mm was used as the substrate material. Mild steel was chosen because it is readily accessible and inexpensive, yet it has inferior mechanical properties compared with stainless steel, despite being the most widely used as industrial steel. The wires used were mild steel (G3Si1) welding wire and austenitic stainless steel (SS304) wire with a diameter of 1.2 mm. Table 2 shows the chemical composition of the wires. The materials were deposited one after another rather than simultaneously. Stainless steel is a highly recommended material for structural applications in nuclear industries, power plants, heavy load engineering applications, high-temperature vessels, construction, and various other industrial uses due to its wide range of mechanical and physical properties. Because of its superior mechanical properties, stainless steel can be used directly in buildings; nevertheless, the cost of such structures is higher due to the high material cost of stainless steel. Stainless steel can be used in structural building with the help of mild steel via dissimilar joining, which produces reasonable results. The stainless steel is corrosion-resistant, and the Ni is added to it, which makes the stainless steel ductile. Some elected properties of mild and stainless steel are presented in Table 3 [21]. Proper metallurgical bonding is required at the interface point when combining these two materials. 

The deposition process was carried out in an argon environment with a purity of 99.99% to prevent the part from oxidizing during the production process. The flow rate was maintained at a level of 14 L/min. The substrate was mechanically polished before being mounted on the machine to remove the oxides and other impurities. 

The impact of the GMAW process factors on the heat input (*HI*) was calculated using the equation:

(1) $$HI=\frac{VI}{S}$$

where *I* is welding current in (Amp), *V* is arc voltage (*V*), and *S* is welding speed (mm/s).

The parameters utilized in the studies are listed in Table 4. As shown in Figure 2, the welding torch is held in the torch holder and motion is controlled by integrated software via the computer. The mild steel substrate was positioned on the CNC worktable. 

In the WAAM process, the temperature rises as a result of continuous metal deposition. This slows the rate of cooling via conduction. The walls began to bulge as a result of the excessive heat build-up. Furthermore, molten metal dripped from the top as the newer layer was deposited on still too hot layers. Therefore, intermittent deposition was used to avoid such situations. After each layer, the part was allowed to cool to 200 °C before the next layer was deposited. This temperature is referred to as the “intermediate temperature” in this work. This method avoided the previously mentioned problems with wall bulging and molten metal dripping [22]. Similar results could be obtained if the deposition path was long enough to allow for a sufficient delay between material depositions at a particular location. However, this situation would only apply to the creation of larger objects. 

Table 4 shows the process parameters for the studies conducted with both materials. The welding speed varied between 5 and 8 mm/s. The current values ranged from 100 to 160 A, and the voltage ranged from 13 to 19 V. The effects of the parameters on bead cross-sections were investigated by depositing one layer for every combination. After determining the optimal variable values, square samples of 60 mm × 60 mm in size were deposited using those variables. Mild steel, stainless steel, and a bimetal of mild steel and SS304 samples were deposited.

### 2.3. Sample Preparation for Metallographic Characterization and Mechanical Testing 

**Figure 3:**
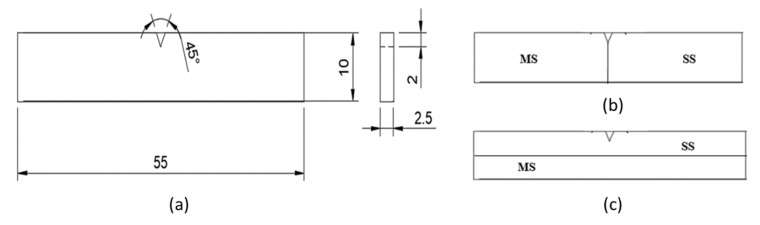
(**a**) Dimensions of nonstandard Charpy sample; (**b**) longitudinal; and (**c**) transverse samples of bimetal.

**Figure 4:**
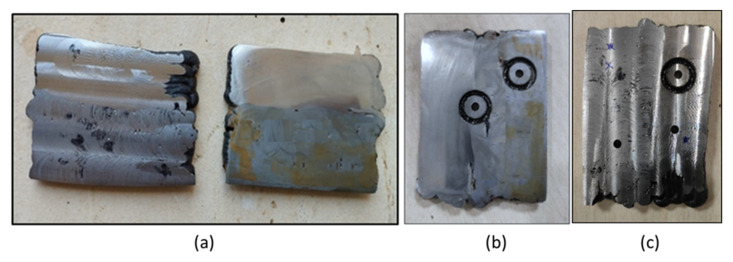
(**a**) Machined surfaces; (**b** and **c**) Holes drilled and trepanned at the interface of the MS and SS portion.

For study of microstructure, samples were cut using an abrasive cutter, and polishing of the samples was performed using a disc polishing machine. The mild steel samples were etched with 2% Nital etchant, and the SS304 samples were etched with V2A etchent. In the case of the bimetallic sample, the mild steel portion was etched first, and the microstructure was observed, followed by etching and microstructure observations of the stainless steel portion. The microstructure of the samples was observed using a Leica DMI3000 M optical microscope (Leica Microsystems GmbH, Wetzlar, Germany). The hardness of the samples was measured using the ZwickRoell ZHVμ Micro Vickers hardness tester ((ZwickRoell GmbH & Co. KG, Ulm, Germany) by applying a load of 100 gf. Hardness was measured along the deposition direction at the center line. To determine the impact strength of the samples, non-standard V notch Charpy samples [23] were prepared as per the dimensions shown in Figure 3. The deep hole drilling (DHD) test was performed to determine residual stress generation in the samples [24,25]. To measure residual stresses, the samples were milled, and a 3 mm reference hole was drilled at three locations: at the interface, in the mild steel portion, and in the stainless steel portion. To relax the strain surrounding the reference hole, the reference hole was trepanned up to a depth of 3 mm using an electro discharge machining (EDM) process, as shown in Figure 4.

Tensile strength was determined by correlating the Rockwell hardness value and strength. Rockwell hardness was determined using a Rockwell hardness machine with a load of 100 kgf and a 1/16" ball indentor. Rockwell hardness was measured at various points in transverse and longitudinal directions to determine the average hardness value.

## 3. Results and Discussion

### 3.1. Effect of Process Parameters on Bead Quality

To optimize the torch’s power and travel speed, the original pilot trials used various combinations of input settings. Table 5 displays the geometric factors, such as width and height, as well as their ocular observations of the deposited tracks. At lower current and voltage, an uneven discontinuous deposition was detected on the material, as shown in Table 5. Regardless of power adjustment, uneven and discontinuous track geometry is generated at lower current and voltage, as shown in Table 5. Because of the high torch travel speed and decreased material availability at the melt pool, there was insufficient energy at the melt pool, resulting in incomplete melting and discontinued and uneven beads. As a result, further investigation of these samples was ruled out. Uneven deposits were also discovered at lower welding power and higher wire feed rate due to an abundance of material in the melt pool at lower power. Beads deposited using optimum parameters are observed to be regular and uniform with uniform width for the entire length. Wire stubbing deposition resulted at a higher power and higher wire feed rate, whereas discontinuous deposition resulted at lower power and lower wire feed rate. As a result, travelling speed is the most influential parameter in the production of geometrical characteristics such as bead width and bead height. The track width varies as a function of travel speed. It was discovered that when travel speed increased, the width of the deposited track shrank. With increased travel speed for a given power and wire feed rate, the melt pool is smaller, resulting in a narrower deposited track. This variation in bead width with travelling speed can be observed from Table 5. It was also observed that deposition height is independent of the process parameters. 

### 3.2. Effect of Shielding Gas on Deposition during Fabrication of the Mild Steel Structure

**Figure 5:**
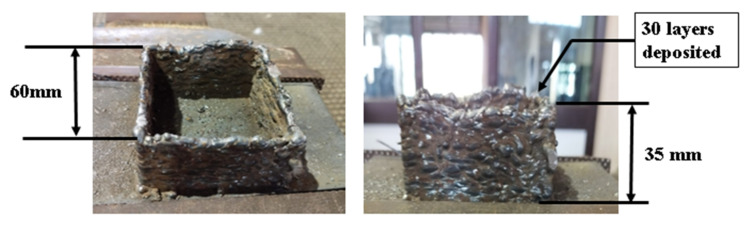
Mild steel sample deposited with CO_2_ shielding gas.

**Figure 6:**
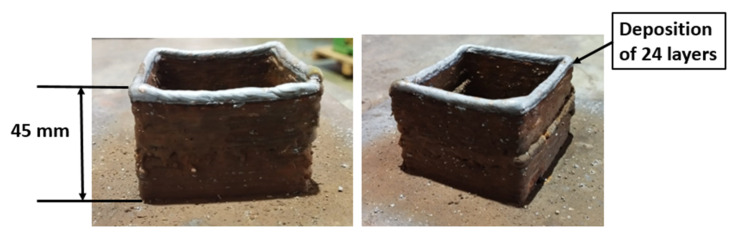
Mild steel sample deposited with argon shielding gas.

Two types of gases, active and inert gases, are commonly used in the GMAW-based AM method. Active gases such as CO_2_ are commonly used in GMAW techniques due to their low cost and ease of availability. Active gases promote oxide production, spatter, and deeper penetration due to the presence of oxygen, which further enhances the localized heat. As a result of the increased heat input, previously deposited layers are reheated, which aids in the refinement of grains and increases the hardness and strength of the manufactured item. Inert gases such as argon (Ar) and helium (He) are becoming more common due to their chemical inertness. As a result, ambient gases such as O_2_, N_2_, and others are unable to react or diffuse within the weld pool, lowering the risk of oxidation and porosity formation. A stable arc can be achieved with the use of inert assist gas, reducing the likelihood of spatter. When the gas flow rate is set high, the heat energy is absorbed by the gas flow, resulting in poor weld pool performance. It is observed that the bead deposited by argon shows an absence of any oxidation, and a bead of uniform width is formed, but when CO_2_ is used, an oxide layer is formed over the surface, and due to increased spatter, the layer is not uniform. In this work, a square structure of mild steel was deposited by the GMAW process, as shown in Figure 5. Two samples were deposited using CO_2_ and argon gas. Using CO_2_ gas, 30 layers were deposited using an inter-pass time of 5 min. The deposition height was measured as 35 mm. However, as discussed in the previous section, irregular deposition occurred because of the higher spatter. Beads with a height of 1.1 mm were deposited, lowering the height of deposition. Apart from the irregular deposition, an oxidation effect was also visible over the surface of the layers. Due to these limitations, another sample was deposited using Ar (Figure 6). With a bead height of 2 mm, there was an improvement in bead quality. A total height of 45 mm was obtained after 24 layers. No oxidation was observed over the surface when using argon gas.

#### 3.2.1. Microstructure of MS 

**Figure 7:**
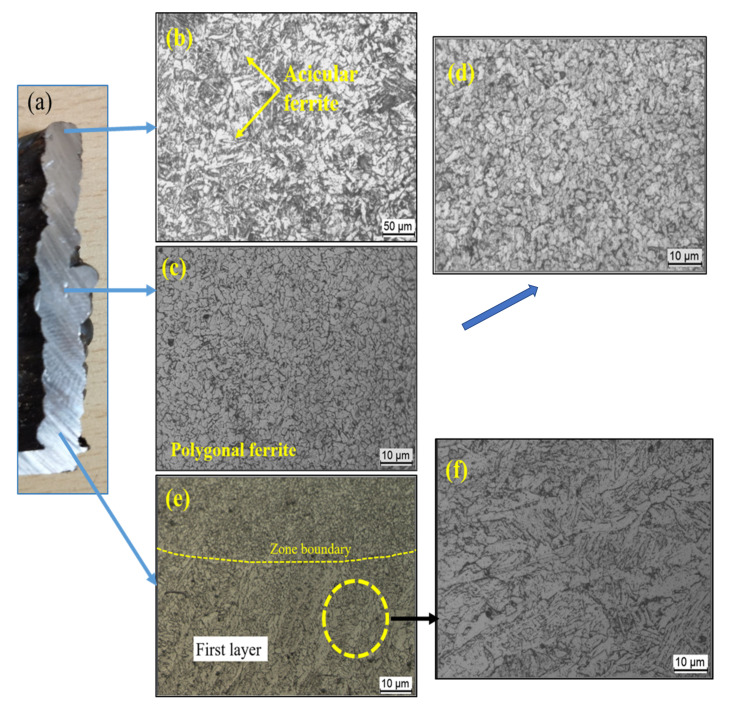
(**a**) Cross-section of the deposited sample; (**b**) microstructure at top layer; (**c**) and (**d**) middle region; (**e**) first layer; (**f**) enlarged view of first layer.

Figure 7 shows the optical microscopy (OM) results of the sample. The cross-section of the deposited sample, with arrows pointing to several locations where the microstructure was examined, is shown in Figure 7a. As the last built layer comes into contact with atmospheric air, it cools faster, creating a bainitic structure with a fine aggregate of α-phase and Fe3C phase, both having an acicular shape. The acicular plates are oriented towards the cooling direction. Because there is no following layer, there is no further heating action on the deposited layer, resulting in a finer grain structure in the last layer. As shown in Figure 7c, the fine ferrite phase (polygonal) dominates the central region, with small regions of pearlite at the grain boundaries. Rapid solidification, which is characteristic of the WAAM process, is the key explanation for the reduced pearlite phase in the WAAM deposited samples. In this location, we can see both coarser and finer grain structures in both overlapped and non-overlapped regions. The molten pool of the current layer reheats and remelts the previously created layer, causing the grain structure to increase, creating coarser grains in overlapping regions [26], as shown in Figure 7d. 

Figure 7e depicts the microstructure of the first layer, with directional columnar grains oriented perpendicular to the substrate (along the depositional direction) and finer formed grains. This could be the result of a higher cooling rate due to thermal conduction to the substrate at room temperature. This higher temperature gradient results in directional solidification. A martensitic phase can be observed in the first layer near the substrate. The presence of martensite in the first layer is due to the instantaneous transformation of the austenite lattice, without the diffusion phenomenon. This transformation from austenite to martensite is due to rapid cooling [27]. As the carbon percentage is less than 0.6%, the formation of lath martensite is expected (Figure 7f).

#### 3.2.2. Hardness of MS

**Figure 8:**
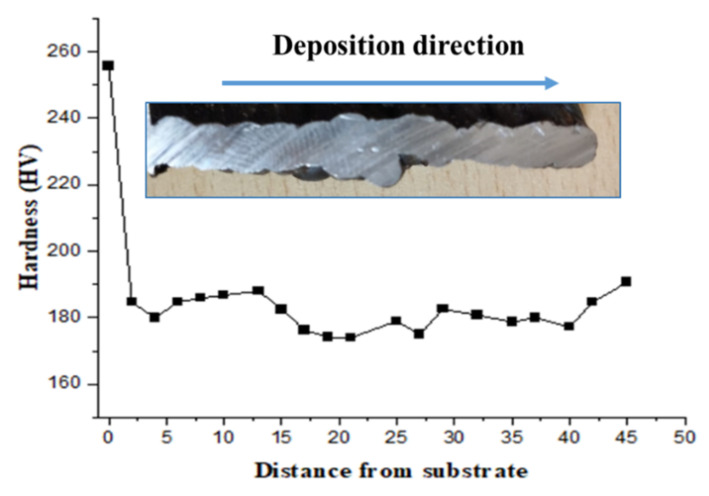
Hardness variation with deposition height in MS.

Figure 8 depicts the hardness characteristics of the mild steel sample. According to the statistics, the heat-affected zone (HAZ) has the highest and lowest hardness value. The HAZ at the fusion boundary has the highest hardness, whereas the HAZ far from the fusion barrier has the lowest hardness. As stated in the previous section, the existence of a martensite phase in the first layer is the cause for increased hardness towards the fusion boundary of the first layer. The variable levels of martensite production discovered at different sites are thought to be the cause of the variance in HAZ hardness. The higher hardness is due to the faster cooling rate of the first few layers. At the commencement of the deposition, the substrate was set at 25 °C, resulting in a substantially faster cooling rate in the first few layers. This layer’s higher hardness could be due to finer grains forming due to the rapid cooling [18]. The cooling rate lowers as more layers are deposited, and due to the reheating effect, coarse grains are formed in the overlapped area, which decreases the hardness as the number of layers increases. The graph shows that the hardness value increases in the last layer, which can be explained by the existence of bainite, whose α - phase is harder than the α - phase in pearlite due to dense dislocations.

### 3.3. Deposition of Stainless Steel Sample

**Figure 9:**
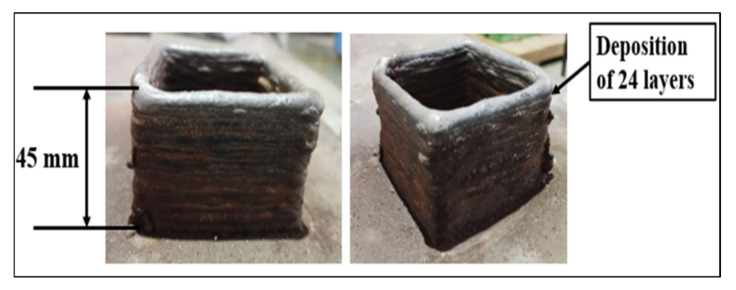
Stainless steel sample deposited with argon shielding gas.

The square structure of stainless steel (SS304) was deposited by the GMAW process, as shown in Figure 9. The use of CO_2_ for SS304 deposition can create chromium oxide, which is not desired in deposited samples. Due to these limitations, the sample was deposited using argon gas. With a bead height of 2 mm, there was an improvement in bead quality. A total height of 45 mm was obtained after 24 layers of deposition.

#### 3.3.1. Microstructure of Stainless Steel

**Figure 10:**
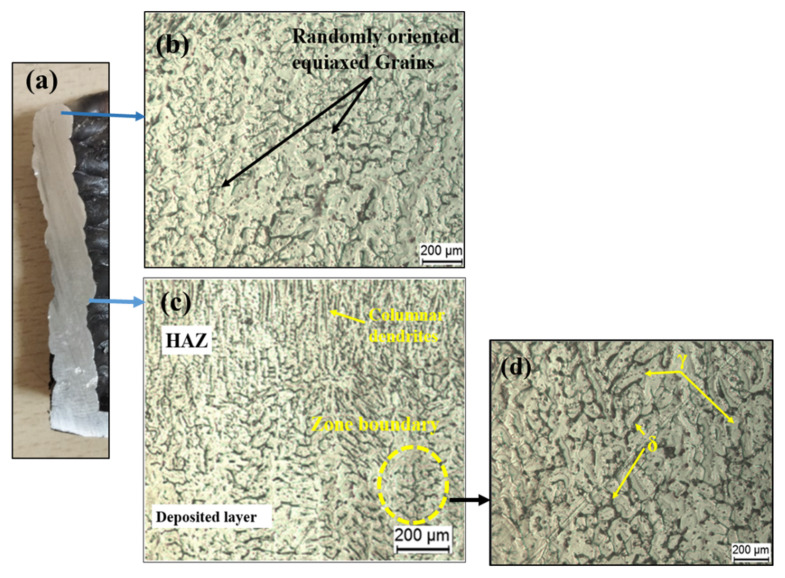
(**a**) Cross-section of deposited sample; (**b**) microstructure at top layer; (**c**) microstructure at interface of two layers; (**d**) enlarged view of HAZ.

Figure 10 shows the OM results of the sample. A cross-section of the deposited sample with arrows pointing to several locations where the microstructure was examined is shown in Figure 10a. There is good bonding between the layers with no internal faults such as pores or micro-cracks. 

Optical microscope images of SS show that it mainly consists of austenite (γ), and delta-ferrite (δ) phases. Considering the Fe–Cr–Ni phase diagram at room temperature, the equilibrium microstructure of 304 is entirely austenitic at 70% Fe [28]. On the other hand, the GMAW method is characterized by strong temperature gradients, high cooling rates, and reheating treatment effects, which results in non-equilibrium microstructures. This effect can be seen in Figure 10. Well-aligned austenitic dendrites are vertically oriented in the sample, generating massive columnar grains in the middle, whereas some dendrites bend towards the plate surfaces, forming small columnar grains towards the edges. Within the austenitic dendrite, the ferrite has a reticular shape. Because of the low C concentration, no carbide appears in the steel. In austenitic steels, the phase transformation is generally as follows [26]:

L → L + δ → L + δ + γ → δ + γ → γ

where L, γ and δ are liquid, delta ferrite and austenite respectively. The δ-phase is the primary phase. Due to rapid cooling and solidification, the austenite phase originates, expands, and gradually replaces the main ferrites. The residual ferrite in an austenite matrix with vermicular or skeletal structures or lathy dendrites is found in the grain and sub-grain boundaries. A higher cooling rate due to the direct conduction of heat from the first layers to the substrate at room temperature results in the formation of finer columnar dendrites growing in various directions.

Figure 10b shows the microstructure at the top layer. It consists of randomly oriented equiaxed grains, which are formed as a result of a slower cooling rate as the top layer comes into contact with the atmospheric air and the absence of a further heating effect. Figure 10c depicts the central region’s microstructure, demonstrating that the δ phase has a skeletal structure within austenitic dendrites. However, as the next layer is deposited, a portion of δ redissolves in γ, and the retained δ exhibits a vermicular shape due to the effects of subsequent heat cycles. The HAZ in Figure 10d depicts the consequence of further heat cycles, in which the vermicular δ phase turns into a strip. The rate of cooling reduces as the number of layers increases, and the direction of heat dissipation becomes visible along the material’s accumulation direction, making the columnar grains appear parallel to each other. Similar results were obtained by Chen et al. [11,29].

#### 3.3.2. Hardness of Stainless Steel

**Figure 11:**
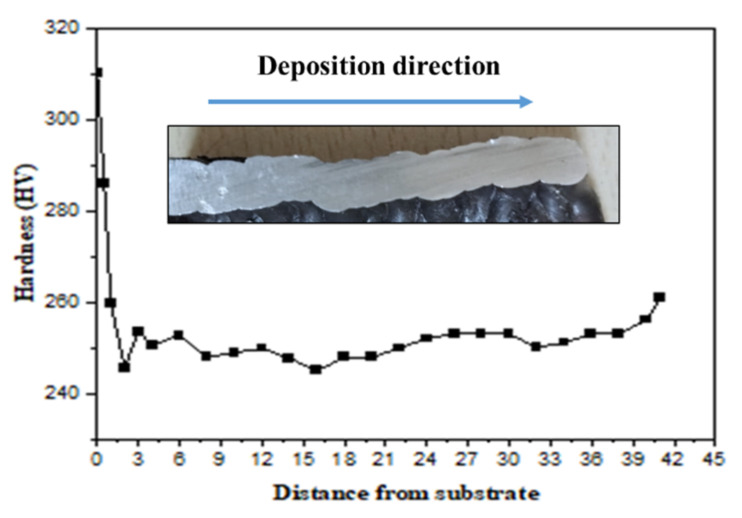
Hardness variation with the deposition height in SS304.

Figure 11 shows the microhardness of GMAW thin-walled 304 measured along the deposition. The observation of microstructures is consistent with the fluctuation of microhardness. Because the microstructure in the bottom section is finer than in the other parts, the microhardness has the greatest average value. The higher cooling rate of the first few layers is responsible for the high hardness. The substrate was set to 25 °C at the start of the deposition, resulting in a significantly higher cooling rate in the first few layers. The higher hardness in this layer could be due to finer grains forming as a result of the rapid cooling.

### 3.4. Deposition of Bimetallic Structure

**Figure 12:**
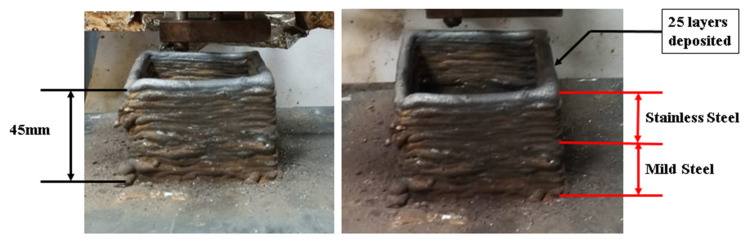
Mild steel and stainless steel bimetallic deposition.

**Figure 13:**
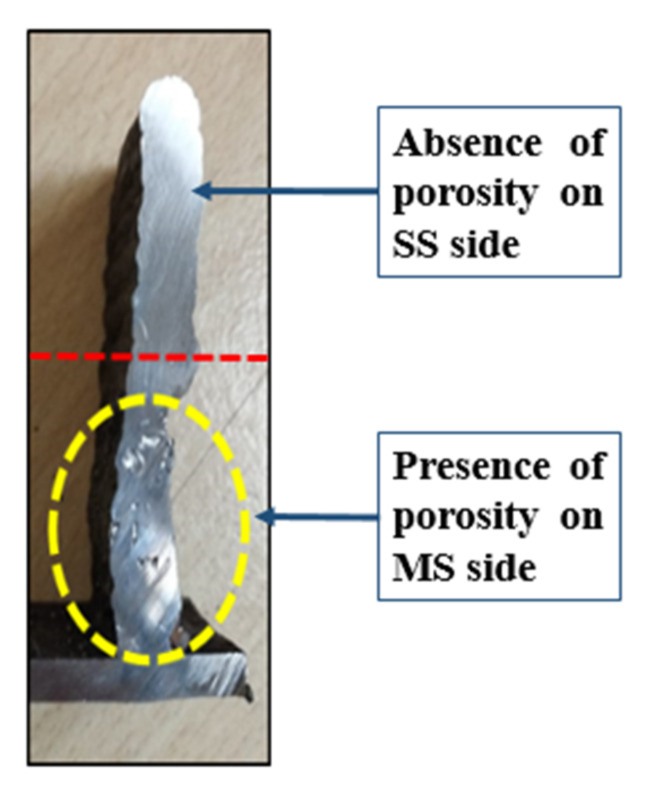
Presence of porosity in the deposited sample.

A square structure of mild steel and stainless steel bimetallic structure was deposited by the GMAW process, as shown in Figure 12. The layers were deposited using an inter-pass time of 5 min after the deposition of every layer. The height of the deposition was 45 mm. Porosity was observed (Figure 13) on the mild steel side; this could be due to the arc blow, resulting in an unstable arc. Because mild steel is a ferromagnetic material, the possibility of an arc blow due to magnetic forces is high; an unstable arc results in increased spatter. On the stainless steel side, no porosity was observed, and the spatter was also very low in comparison to that of the mild steel; this could be due to the nonmagnetic nature of SS 304. The shielding gas pressure is important in the formation of porosity; as the gas pressure decreases, the amount of porosity increases. Another sample was deposited with the optimal shielding gas pressure and a controlled process (removing the metal stuck to the torch nozzle due to spatter to prevent arc blow).

#### 3.4.1. Microstructure of Bimetallic Structure

**Figure 14:**
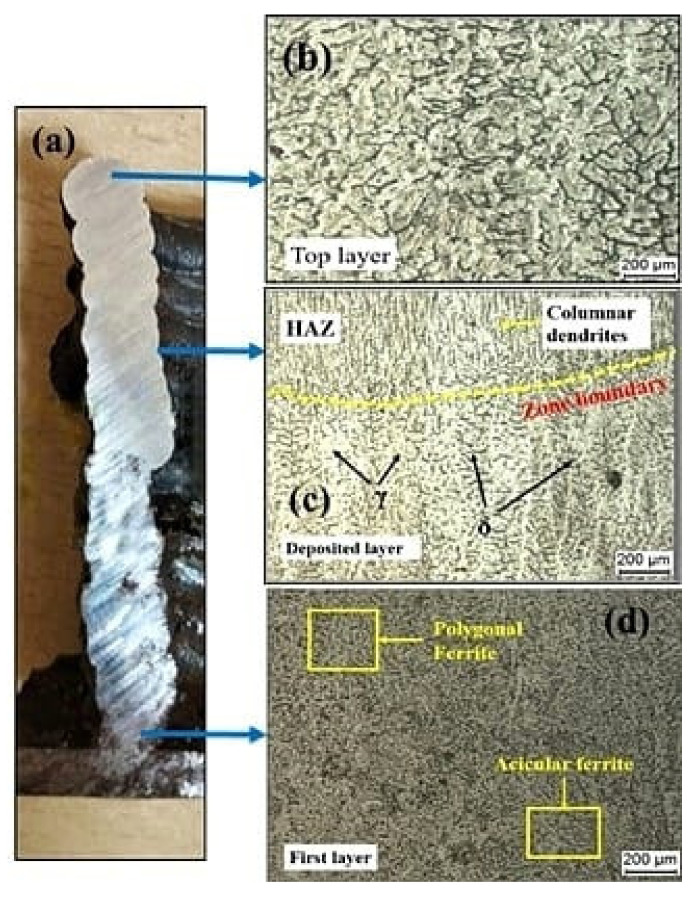
(**a**) Cross-section of bimetal deposition; (**b**), (**c**), (**d**) microstructure at indicated locations.

**Figure 15:**
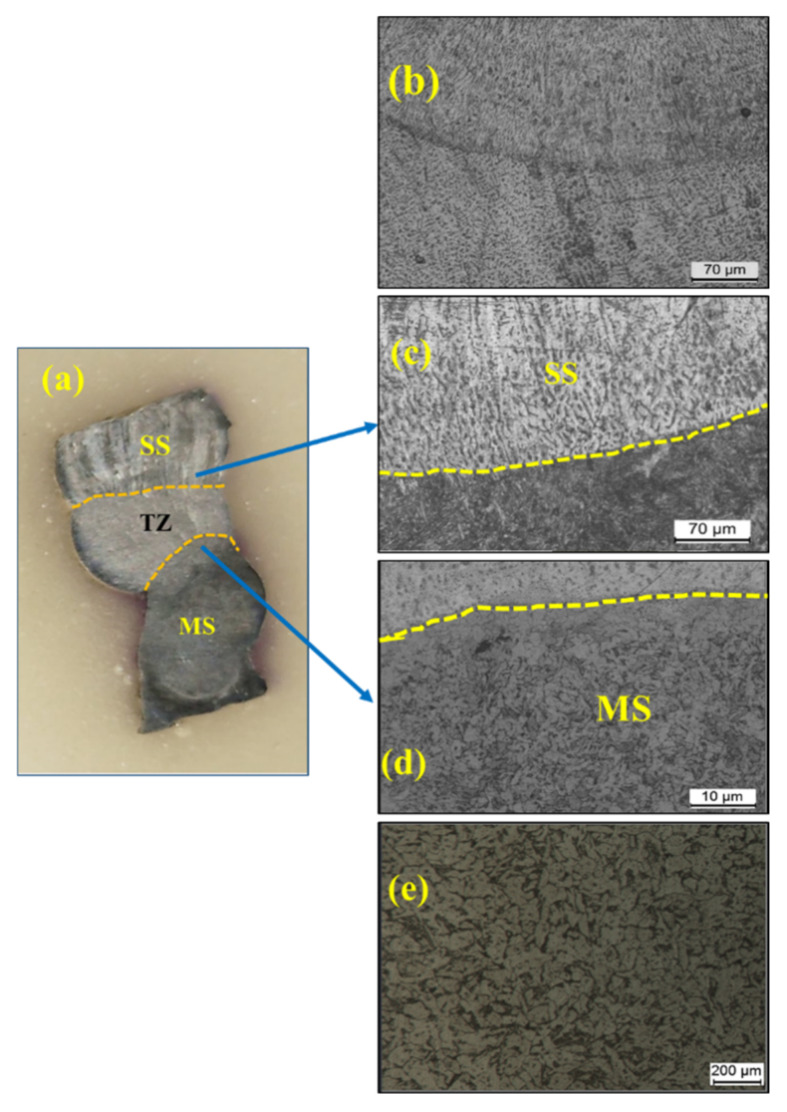
(**a**) Intersection of MS and SS304; (**b**) microstructure of SS304; (**c**), (**d**) microstructure at indicated locations; (**e**) microstructure of MS.

Figure 14 shows the microstructure variation in the bimetallic sample along the deposition direction. Similar microstructure variation was observed in the top layer and first layer as in the stainless steel and mild steel samples, respectively, as discussed in the previous sections. The region specified by Figure 15e is the interface region of MS and SS. A cross-section of the interface is shown in Figure 15, with two distinct regions visible. The images reveal a heterogeneous microstructure at the interface. The microstructure reveals columnar grains with strong anisotropy toward the build direction on the as-deposited SS304 side (Figure 15b,c). This is due to the fact that the cooling is directional. Ferrites in the austenite matrix are microstructural features that point to a ferrite to austenite (FA) transformation [18]. The FA transformation produces a skeletal ferrite and lath morphology with austenite and ferrite. However, at the interface, no defects such as cracks or porosity are visible. On the MS side, the microstructure in Figure 15d shows the presence of the ferrite phase with the presence of pearlite at the grain boundaries. The grains are enlarged and coarse grains are formed as a result of increased heat input and repeated thermal cycles.

#### 3.4.2. Hardness of Bimetallic Structure

**Figure 16:**
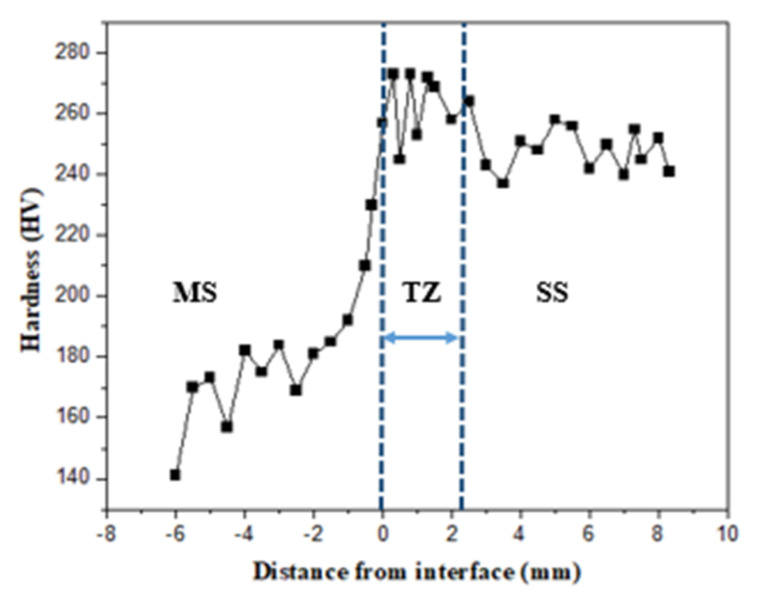
Hardness variation along the deposition height at the interface.

**Figure 17:**
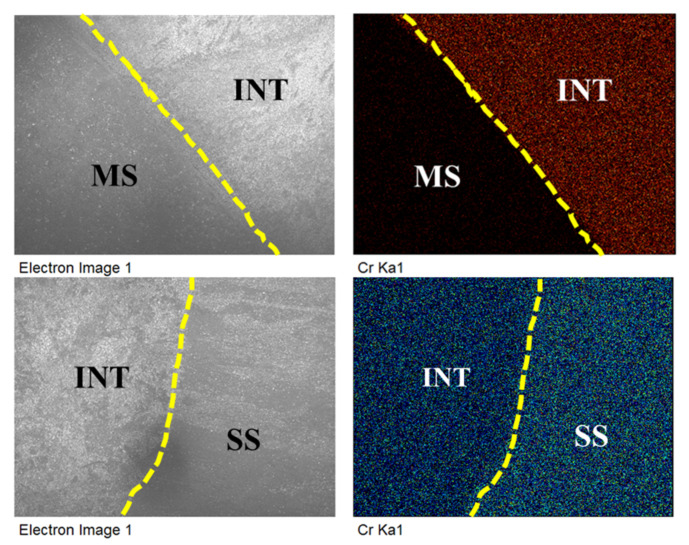
Elemental mapping images at interface.

The hardness profile shown in Figure 16 shows a sharp increase in hardness value near the interface. From 180 to 280HV, the hardness value rises. The migration of chromium into the MS side is responsible for the increase in hardness at the interface. The migrated chromium may cause a solid solution hardening effect, increasing the hardness [18]. The results are also supported by energy dispersive spectroscopy (EDS) analysis. Figure 17 shows the elemental mapping images at the interface of the bimetal sample on both the SS304 side and the mild steel side. The higher chromium percentage is seen on the SS304 side and propagates to the mild steel side.

### 3.5. Charpy Test

**Figure 18:**
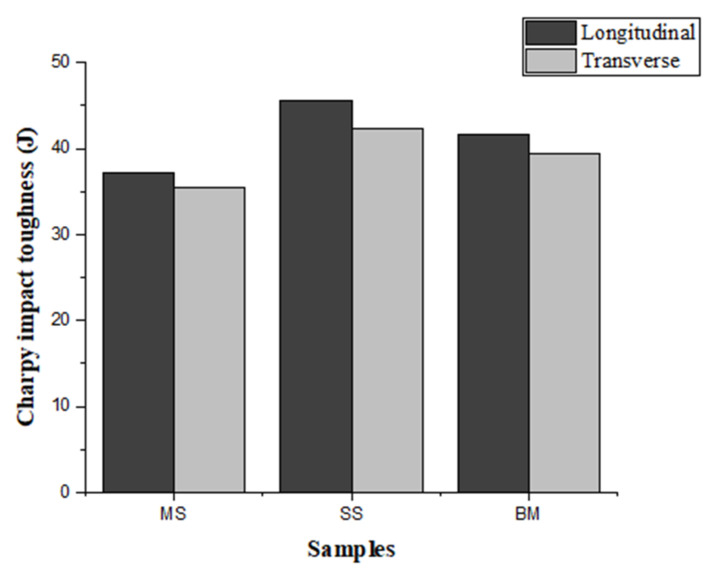
Comparison of Charpy results in the transverse and longitudinal direction.

**Figure 19:**
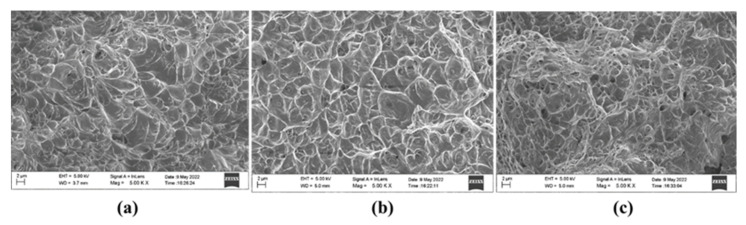
Fractured surface of (**a**) mild steel (**b**) stainless steel (**c**) bimetal.

Charpy V sub-sized test samples were prepared in transverse and longitudinal directions. Figure 18 shows the Charpy test results of the samples tested in both directions. The Charpy impact toughness was higher in the longitudinal direction, which could be due to grain growth in the AM components along the cooling direction and results in good bonding between the layers (Figure 18). The impact toughness of SS304 was higher than that of mild steel and bimetal. The impact toughness of bimetal was between those of mild steel and SS304. In the longitudinal direction, due to good bonding at the interface, the impact toughness was increased. In the transverse sample, the breakage initiated in the mild steel, causing a reduction in impact toughness. Even though there was a difference in toughness values in the longitudinal and transverse directions, it was so small that we can conclude that the Charpy toughness is found regardless of testing direction. The fracture surfaces exhibited a typical pattern for ductile materials (Figure 19). Because the fracture surface of the specimens was not shinier and comprised grooves and dimples, we can conclude that it was a ductile failure [30,31].

### 3.6. Tensile Strength Using Rockwell Hardness

Studies by Hui et al. [32] show the conversion of hardness and hardness-strength conversion calculated from the theoretical equation. A satisfactory match was observed when the calculated and standard values were compared. It was observed that the strength values obtained by Rockwell hardness (HR) result in a better fit with the standard values than those produced by Vickers (HV) and Brinell hardness (HB). Thus, using Rockwell hardness data to determine the sample’s tensile strength is advantageous. The hardness variation trend was similar to that of Vickers harness. The hardness values were converted into tensile strength as per the conversion table given in [33] which is produced from ASTM A370.

For converting the Rockwell hardness to tensile strength, equations proposed by Petrenko [34,35] can also be used.

(2) $$\mathrm{Tensile}\;\mathrm{strength}\;(\mathrm{lbs}/{\mathrm{in}}^{2})=\frac{3760000}{130-\mathrm{HRB}}\;\;\;\;\mathrm{for}\;\mathrm{HRB}\;\mathrm{less}\;\mathrm{than}\;88$$

(3) $$\mathrm{Tensile}\;\mathrm{strength}\;(\mathrm{lbs}/{\mathrm{in}}^{2})=\frac{3580000}{130-\mathrm{HRB}}\;\;\;\;\mathrm{for}\;\mathrm{HRB}\;\mathrm{greater}\;\mathrm{than}\;88$$

**Figure 20:**
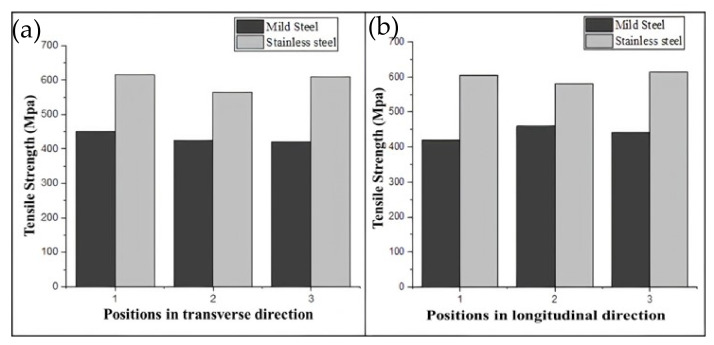
Tensile strength variation in (**a**) transverse and (**b**) longitudinal directions in mild steel and stainless steel.

**Figure 21:**
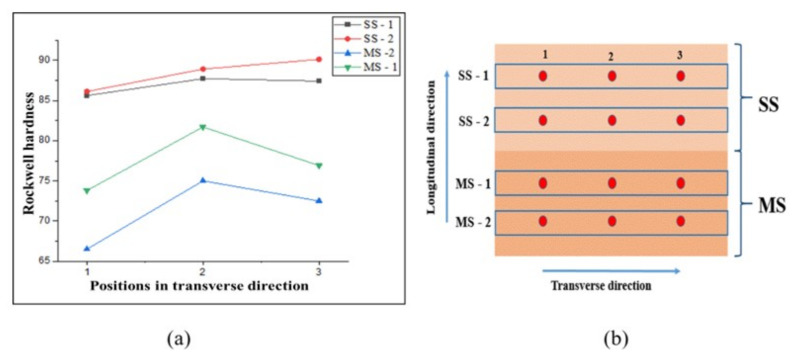
(**a**) Hardness variation in transverse direction at various sections in the bimetal sample, (**b**) Positions where the hardness was measured.

Table 6 shows the tensile strength and average hardness of mild steel and stainless steel in transverse and longitudinal directions at three different places. Variance in tensile strength values was observed at the different locations, which can be explained as the result of thermal history variation. In transverse and longitudinal directions, average tensile strengths of approximately 432 MPa and 440 MPa, respectively, were recorded, which are slightly greater than the tensile strength of MS G3Si1 welding wire (379–483 MPa), according to ASTM specifications. In the SS samples, there was a similar fluctuation in tensile strength with regard to position. Average tensile values of approximately 597 MPa and 600 MPa were measured in transverse and longitudinal directions, respectively, which are slightly higher than the tensile strength of SS 304 welding wire (515 MPa), according to ASTM requirements. Anisotropy in properties is observed in the WAAM deposited samples. This variance can be attributed to grain expansion along the deposition direction as a result of directional cooling in AM. The strength of the SS sample is greater than that of the MS and bimetallic samples, as shown in Figure 20. Similar results were obtained by Haden et al. [36]. At the interface of the bimetallic samples, tensile strengths of 485 and 550 MPa were measured in the transverse and longitudinal directions, respectively. The bimetallic sample had a tensile strength halfway between those of SS and MS. In the bimetallic samples, it was difficult to locate the exact failure position. SS304 has a lower yield strength than mild steel so we can speculate that the failure may have initiated in the SS portion. The hardness variation along the transverse direction at several locations along the deposition length in the bimetallic sample is depicted in Figure 21. The graph shows that the hardness values are higher in the SS position than in the MS position and at the interface of the two metals there is an increase in the hardness.

### 3.7. Residual Stress Analysis

**Figure 22:**
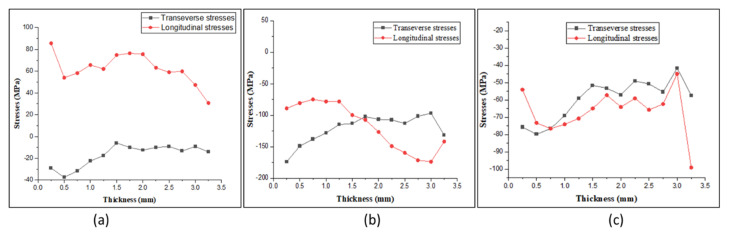
Residual stress distribution through thickness on (**a**) SS304, (**b**) interface, and (**c**) mild steel.

Deep hole drilling (DHD) is a semi-destructive method used for measuring the through-thickness residual stresses based on strain relaxation methodology. This method estimates the locked-in stresses from the deformation of a reference hole following material removal [37,38,39]. The DHD process mainly involves four steps: (1) drilling of a reference hole; (2) measuring the hole diameter by an air probe; (3) trepanning the reference hole using EDM; and (4) re-measuring the hole diameter by an air probe [40,41]. Figure 22 shows the residual stress distribution throughout the thickness at three positions along the height of deposition, one on mild steel, one at the interface and one on the stainless steel portion, as shown in Figure 4 in Section 2. Longitudinal stresses are stresses generated in the torch moving direction, and transverse stresses are perpendicular to the torch direction. The graphs show that the longitudinal stresses were more dominant than transverse stresses at all positions. In the SS304 section, the longitudinal residual stresses were observed to be tensile in nature, and the transverse residual stresses were observed to be compressive. Peak longitudinal stresses of 90 MPa were observed; however, the longitudinal stresses were observed in the range of 60 to 80 MPa. The peak magnitudes of transverse residual stresses were observed to be 40 MPa. However, at the interface of SS and MS, the residual stresses were observed to be compressive in nature. It can be seen that highly compressive residual stresses were observed at the interface. At the outer surface of the interface, the longitudinal residual stresses were observed to be at 90–100 MPa, reaching a peak magnitude of about 195 MPa. As the deposited sample is square, the cooling rate differs on the outer and inner faces. From the graphs, we can see that the residual stresses varied throughout the thickness, with higher stresses at the outer face that towards the inner surface. As the outer face comes in contact with the air, the cooling rate is higher and the grains formed are finer, but on the inner side, the cooling rate is slower due to decreased airflow, resulting in coarser grains. This variation in grain size could be the reason for the variation in residual stresses through the thickness. In the case of SS304 (top layer), the longitudinal stresses generated were tensile in nature due to higher constraints to the metal shrinkage from the already solidified metal in the longitudinal direction. The transverse stresses are compressive in nature, as there is no constraint in the transverse direction for metal shrinkage. However, with residual stresses at the interface and mild steel being compressive in nature, these stresses can arise due to the reheating effect on already deposited layers. When a new layer is deposited over the sublayer, the metal tries to expand, resulting in the generation of compressive stresses. On the SS304 side, as it is the top layer deposited, there is no reheating effect over the deposited layer, which is why the stresses are tensile. The stresses generated are less than the yield strength of the material. Compressive residual stress can positively impact fatigue performance because it acts to resist the applied tensile stress as it holds the crack faces shut, minimizing damage [42].

## 4. Conclusions

In the present study, an experimental investigation was carried out to fabricate a 3D geometry based on the WAAM process using the GMAW process. 3D printing was successfully modified and used for the application. The effect of heat input on the distribution of residual stresses after fabrication was determined using the deep hole drilling method. From the present study, it can be concluded that GMAW can be effectively used for the fabrication of 3D geometry with considerable hardness and strength. The fabrication using GMAW WAAM of complicated structures, such as the gears and guide vanes of turbines, are possible future applications.

1. A wood engraving machine was successfully transformed into a metal 3D printer for the WAAM process in this work.
2. The mechanical properties, microstructure, and composition of the bimetallic specimen were investigated and compared with the mechanical properties of a single MS and SS material specimen. The hardness was observed to be higher in SS section in both the longitudinal and transverse directions and was in the range of 240–260 HV. However, in the MS section the hardness was in the range of 160–180 HV. The diffusion of chromium from the SS side into the MS is responsible for the increased hardness.
3. There were no welding defects at the two-material contact. This research indicates that using the WAAM technique, MS and SS can be successfully merged without any defects.
4. The samples’ tensile strength was calculated using Rockwell hardness values. The MS and SS tensile strength were found to be slightly higher than the conventional values. The tensile strength of bimetal was between that of the MS and SS.
5. The residual stresses created in the bimetallic sample were determined using DHD. Both the longitudinal and transverse residual stresses were observed to be compressive in nature with a range of 50–80 MPa in the mild steel section, whereas in the SS section, the longitudinal stresses were tensile in nature, with a value of 90 MPa observed at the surface. The transverse residual stresses were found to be compressive, with a value of 30 MPa at the surface.

## Figures and Tables

**Table 1:** Chemical composition of 6061 aluminum alloy.

Components	Mg	Si	Fe	Cu	Zn	Ti	Mn	Cr	Al
Percentage	0.8–1.2	0.4–0.8	0.7	0.15–0.40	0.25	0.15	0.15	0.04–0.35	Balance

**Table 2:** Chemical composition of substrate and wire.

Steel	C	Si	Mn	Ni	P	S	Cr	Mo	Al
Substrate (S235JR)	0.2	0.55	1.4	0.012	0.045	0.04	0.3	0.08	0.02
Mild steel (G3Si1)	0.15	0.25–0.75	0.2–0.5	0.65	0.07–0.15	0.03	0.5–1.25	0.001	0.015–0.06
Stainless steel (304)	0.07	1.00	2	8.00–10.50	0.045	0.015	17.50–19.50	-	-

**Table 3:** Properties of mild steel and stainless steel.

Properties	Mild Steel	Stainless Steel
Thermal conductivity (W/mK)	45	16.2
Specific heat (J/kgK)	450	502.416
Thermal expansion coefficient (10^−6^/°C)	10	17.3
Young’s modulus (GPa)	200	193
Density (kg/m^3^)	7900	8000
Yield strength (N/mm^2^)	250	215
Poisson’s ratio	0.2786	0.275
Melting point (°C)	1350–1420	1400–1450

**Table 4:** Process parameters for mild steel and stainless steel.

Parameter	Mild Steel	Stainless Steel
Arc voltage (V)	13–17	19
Welding current (A)	100–130	160
Travel speed (mm/s)	5–8	5–8
Diameter (mm)	1.2	1.2
Shielding gas type	argon	argon
Shielding gas flow rate (L/min)	14	14
Substrate thickness (mm)	3	3

**Table 5:** Combination of parameters and outcomes.

No.	Arc Voltage (V)	Welding Current (I)	Travel Speed (mm/s)	Shielding Gas	Bead Width (mm)	Bead Height (mm)	Observation
1	25	240	8	Ar	8.52	1.1	Regular deposition
2	25	160	8	Ar	6.9	1.2	Regular deposition
3	25	130	7	Ar	7.2	1.1	Regular deposition
4	19	170	5	CO_2_	5.5	1.2	Regular deposition
5	18	160	5	CO_2_	4.8	1.3	Regular deposition
6	15	130	5	CO_2_	4	1.5	Discontinuous deposition

**Table 6:** Average hardness and predicted tensile strength.

Direction		Mild Steel			Stainless Steel			Bimetal
Position		1	2	3	1	2	3	Interface
Transverse	Hardness (HRB)	74	71	71	91	85	89	79
	Predicted Tensile Strength (MPa)	450	425	420	615	565	610	485
Longitudinal	Hardness (HRB)	70	76	69	89	88	91	82
	Predicted Tensile Strength (MPa)	420	460	440	605	580	615	550

## Data Availability

Not applicable.
